# Embryonic perfect repair inspired electrospun nanofibers dressing with temperature-sensitive and antibacterial properties for wound healing

**DOI:** 10.3389/fmicb.2023.1233559

**Published:** 2023-07-14

**Authors:** Qinbing Qi, Rongkai Li, Chunhua Wang, Guige Hou, Chengbo Li

**Affiliations:** Key Laboratory of Medical Antibacterial Materials of Shandong Province, School of Pharmacy, Binzhou Medical University, Yantai, China

**Keywords:** wound healing, electrospinning, antibacterial, temperature-sensitive, quaternized silicone

## Abstract

**Introduction:**

The development of highly effective wound dressings is crucial for successful clinical applications. Achieving wound closure, preventing infection, and minimizing scarring are key objectives in wound healing. Drawing inspiration from the regenerative mechanisms observed in embryonic tissue repair, we designed a series of wound-contractible dressings with exceptional antibacterial properties.

**Methods:**

This was achieved by encapsulating quaternized silicone (QP12) and poly(*N*-isopropylacrylamide-co-*N*-hydroxymethylacrylamide-co-octadecyl acrylate) (PNNS) within electrospun nanofibers of poly(ε-caprolactone) (PCL).

**Results and discussion:**

The resulting nanofibrous dressings demonstrated remarkable thermo-responsive self-contraction and tissue adhesion capabilities, enabling secure adherence to the skin and active wound closure. Notably, these nanofibers exhibited potent antibacterial activity against both Gram-positive and Gram-negative bacteria. Furthermore, they possessed desirable properties such as hydrophilicity, biocompatibility and mechanical properties resembling human skin. A full-thickness skin defect model evaluation revealed that these temperature-sensitive nanofibers expedited wound closure, enhanced wound healing, and suppressed scar formation. This result was evidenced by reduced infiltration of inflammatory cells, well-organized collagen arrangement, and improved vascularization. In summary, we propose that these wound-contractible nanofibers, with their antibacterial and anti-scarring properties, hold great promise as an advanced solution for skin wound repair.

## 1. Introduction

The human skin serves as a natural protective barrier against various external threats, safeguarding internal tissues and organs from physical, mechanical and microbial assaults (Bouwstra and Ponec, 2006; Gravitz, 2018). However, the skin is vulnerable to external damage from burns, cuts and bruises, which can give rise to severe health issues and bacterial infections (Del Gaudio et al., 2020). Consequently, the development of wound dressings that effectively prevent cutaneous wound contamination is of utmost importance. Electrospun nanofibers have emerged as promising materials for wound dressings due to their high specific surface area, extensive porosity, structural similarity to the extracellular matrix, excellent biocompatibility and hemostatic properties (Kerwald et al., 2022; Zhong et al., 2023). Additionally, electrospinning facilitates the incorporation of various functional ingredients into the fibers, enabling the creation of novel fibrous dressings with multifunctional capabilities. To expedite wound healing, substantial efforts have focused on the delivery of growth factors and antibiotics through electrospun nanofibers (Glges et al., 2022; Hsiung et al., 2022; Yuan et al., 2022). Unfortunately, these established approaches are plagued by drawbacks such as drug side effects and challenges in achieving controlled release. Hence, there is a compelling need for an active approach to wound healing that circumvents drug side effects and intricate fabrication processes. The remarkable ability of skin injuries in embryos to heal rapidly, effectively and flawlessly without external intervention has inspired researchers to explore the underlying mechanisms governing embryonic tissue repair. Such investigations have yielded novel therapeutic strategies aimed at enhancing wound healing in adults (Nodder and Martin, 1997; Redd et al., 2004).

The process of wound closure in both embryonic and adult tissues involve a combination of connective tissue contraction and re-epithelialization movements. However, the mechanisms underlying these movements differ significantly (Redd et al., 2004). In adult wounds, connective tissue contraction is orchestrated by specialized contractile myofibroblasts, whereas in embryonic epidermal wounds, actin cables generate force to bring the wound edges together in a purse-string-like fashion (Grinnell, 1992; Brock et al., 1996; Werner and Grose, 2003). Another notable distinction between adult and embryonic tissue repair is the extent of inflammation during healing. Adult cutaneous wounds typically elicit a pronounced inflammatory response, whereas inflammation is minimal in embryonic wounds. Insights gained from studying the repair processes of adult and embryonic tissues have provided valuable inspiration, highlighting the importance of active wound closure and limited inflammation for optimal healing (Redd et al., 2004). Moreover, scar formation must be minimized to facilitate immune system activation and extracellular matrix remodeling, both of which play crucial roles in promoting tissue regeneration in adults (Ahn et al., 1991; Julier et al., 2017; Tang et al., 2022). Through studies of embryonic repair, mechanical forces do play a key role in wound inflammation onset and scar size. In early embryonic development, inflammation and scarring do not occur due to the absence of platelets and inflammatory cells involved. This has been shown in clinical studies to suppress scarring through both inflammation suppression and tension reduction techniques.

In order to enhance adult wound healing by emulating the regenerative capabilities of embryonic tissues, a strategy involving the incorporation of polymers with diverse functionalities into electrospun nanofibers has been employed. Firstly, a temperature-responsive polymer has been utilized to restore the wound contraction ability of adult skin. This temperature-responsive dressing exhibits active contraction in response to the body’s temperature, thereby facilitating the contraction of the surrounding tissue in the wound area (Li et al., 2022). Additionally, the quaternized silicone (QP12) polymer, previously reported in our work (Wang et al., 2016; Gao et al., 2023), has been incorporated to augment the antibacterial and anti-scarring properties of the dressing. QP12 combines the anti-scarring effects of silicone with the antibacterial activity of quaternary ammonium salt. In this study, we have successfully developed a mechanically active nanofibrous wound dressing with temperature-sensitive properties. This dressing incorporates the desirable mechanical characteristics of poly(ε-caprolactone) (PCL), the antibacterial and anti-scarring properties of quaternized silicone (QP12), and the temperature-responsive properties of poly(*N*-isopropylacrylamide-co-*N*-hydroxymethylacrylamide-co-octadecyl acrylate) (PNNS). The resulting biomimetic dressing demonstrates multifunctional attributes, including antibacterial activity, hemostasis and scar inhibition, thereby holding significant promise for applications in wound healing.

## 2. Materials and methods

### 2.1. Materials

1,1,1,3,3,3-Hexafluoro-2-propanol (HFIP, 99.5%), *N*-isopropylacrylamide (NIPAm, 98%), 2,2′-Azobis(2-methylpropionitrile) (98%), and Tetrahydrofuran (AR, 99.0%) were supplied by Shanghai Macklin Biochemical Co., Ltd. (China). Octadecyl acrylate (SA, ≥97%), *N*-(Hydroxymethyl) acrylamide (NMA, ≥98%) were purchased from Shanghai Haohong Biotechnology Co., Ltd. (China). Penicillin-Streptomycin Liquid (100 ×) and Trypsin-EDTA solution were provided by Beijing Solarbio Science and Technology Co., Ltd. (China). Phosphate Buffer Saline (PBS) and powder (7.2–7.4) were provided by Servicebio Biotech, Co., Ltd. (China). Polycaprolactone Standard (PCL, MW 80000) was from Shenzhen Esun Industrial Co., Ltd. (China). DMEM (high glucose) were obtained from Cytiva Co., Ltd. (American). The group synthesized quaternized silicones (QP12) by silica-hydrogen addition reaction of polymethylhydrosiloxane in the early stage.

### 2.2. Synthesis and characterization of thermosensitive polymer (PNNS)

Poly(*N*-isopropylacrylamide-co-*N*-hydroxymethylacrylamide-co-octadecyl acrylate) (PNNS) terpolymer was synthesized through radical copolymerization using NIPAm, NMA, and SA as copolymer monomers. Briefly, the synthesis procedure involved adding 9.05 g NIPAm, 1.62 g NMA, 0.81 g SA, and 20 mL THF to a three-necked flask equipped with reflux condenser. The mixture was stirred for 20 min under a nitrogen atmosphere, and then 10 mg of AIBN was introduced. Sequential bubbling under nitrogen for 30 min was performed to remove oxygen. Polymerization was conducted at 65°C for 24 h. The resultant polymer was purified by reprecipitation from 50 mL of THF into 150 mL of n-hexane two times, followed by drying in a vacuum oven for 72 h.

The nuclear magnetic resonance (^1^H NMR) spectra of PNNS were acquired using a Bruker AV 600 MHz spectrometer with deuterated dimethyl sulfoxide as the internal reference. Fourier transform infrared (FTIR) spectra were obtained using an Infrared detector (Nicolet is50, Thermo fisher, America) with a scan range of 4,000–400 cm^–1^ and a resolution of 4 cm^–1^. The absorption of PNNS solution at 500 nm was measured on UV-visible photometer (DS-11FX+, DeNovix, America) to determine Lower Critical Solution Temperature (LCST).

Molecular weights (Mw and Mn) and molecular weight distributions determined by gel permeation chromatography (GPC) measurements on a Waters LS measurement system (Agilent1260, America) with tetrahydrofuran (THF) as the solvent. The flow rate was 1.0 mL/min, and the column temperature was 30°C. The molecular weight distribution was calibrated with standard polystyrene samples.

### 2.3. Fabrication of electrospun nanofibrous membrane (NQP)

First, the composite of QP12/PCL at a weight ratio of 30:70 was dissolved in HFIP. Then, 2% of PNNS was added to the mixed solution and the total concentration of polymer was 8% (w/v). Subsequently, the prepared spinning solution was injected into a 10 mL standard plastic syringe equipped with a standard blunt end needle (20 G, Φ 0.6 mm) with a flow rate of 1.2 mL/h. The applied voltage was controlled at 19 kV. The nanofibers were collected on an aluminum foil paper wrapped in a roller of 15 cm from the syringe needle tip and vacuum-dried for 24 h to completely consume residual organic solvent. Electrospinning was performed at 20 ± 2°C and an average humidity of 35 ± 2%. Four kinds of QP12/PNNS/PCL electrospun nanofibers were synthesized and named as NQP-1, NQP-2, NQP-3, and NQP-4 with different contents of PNNS of 1, 2, 3, and 4%. Electrospun nanofibers without PNNS were prepared as controls (MQP).

### 2.4. Fiber characterization

#### 2.4.1. Scanning electron microscopy (SEM) observation

The surface morphology of the fibers was analyzed with a scanning electron microscopy (EVO LS15, Zeiss, Germany). Samples were gold sputter coated for 60 s under argon to render them electrically conductive before visualization. The average fiber diameter for each sample was calculated by measuring approximately 200 fibers in SEM images using the Image J software.

#### 2.4.2. Water contact angle measurements

The water contact angle (WCA) was determined on a contact angle analyzer (SZ-CAMD33, Shanghai Sunzern Instrument Co., Ltd., China). A water droplet (ca. 10 μL) was placed onto the surface of the fibers and the WCA was recorded after 1 min. Each experiment was repeated three times, and the results were given as average.

#### 2.4.3. Water vapor transmission rate

A 10 mL centrifuge tube was filled with 9 mL of 0.01 M PBS and weighed. The weight of experimental group was denoted as W_0_ and the control group as W_1_. Each dressing was fixed onto the opening of centrifuge tube. The centrifuge tube was placed in an oven at 37°C for 24 h and weighed again. The weight of experimental group was denoted as W_2_ and the control group as W_3_. The control group didn’t cover covering dressing. Each experiment was repeated three times, and the results are given as average. The WVTR was calculated using the following equation:


$$\text{WVTR}\left(\%\right)=\frac{{\text{W}}_{0}-{\text{W}}_{2}}{{\text{W}}_{1}-{\text{W}}_{3}}\times 100\%$$

#### 2.4.4. Water uptake capacity

To estimate the capacity of the wound dressings to absorb wound exudates, the water uptake capacity and water retention capacity were calculated as followed. Briefly, the dry dressings were weighed (W_0_) followed by immersion in PBS at room temperature for 24 h. The fluid-filled dressings were subsequently gently transferred to a Petri dish, where the dressings were allowed to stand for 1 min to allow the removal of excess liquid on the surface. The dressings were then weighed as W_1_. Subsequently, the samples were centrifuged at 500 rpm for 3 min and weighed (W_2_) to calculate the water uptake ability and water retention capacity from the following equation. Each experiment was repeated three times, and the results were given as average.


$$\text{Water}\;\text{Uptake}\;\text{Rate}=\frac{{\text{W}}_{1}-{\text{W}}_{0}}{{\text{W}}_{0}}$$

$$\text{Water}\;\text{Retention}\;\text{Rate}=\frac{{\text{W}}_{2}-{\text{W}}_{0}}{{\text{W}}_{0}}$$

#### 2.4.5. Mechanical properties measurements

Mechanical properties of the samples were determined by universal testing machine (CMT8502, MTS Systems (China) Co., Ltd., America) at a crosshead speed of 10 mm/min. All the tests were performed at 23–25°C and a humidity of 40–50% following standard test method ASTM D-638. Standard dumbbell nanofiber samples with dimensions of 20 mm × 2 mm were used. Each experiment was repeated three times, and the results were given as average.

#### 2.4.6. Adhesion performance

Porcine skin samples were cut to 90 mm × 30 mm and kept in water prior to use. The electrospun nanofibers were cut to 20 mm × 20 mm and placed on the skin samples’ surface. The specimens were adhered to the porcine skin before the experiment, then the adhesion properties were determined by load bearing test.

#### 2.4.7. Thermo-responsive properties

The nanofibers were cut into rectangle pieces 2 cm × 2 cm and placed into a glass vials. Then 20 mL of distilled water (DW) was added. The nanofibers were treated for 10 min at 20, 38, and 40°C, respectively. After fully swelling, photographs of the nanofibers were taken by setting 112 pixels as 1 cm.

### 2.5. Antibacterial activity

In the assessment of antibacterial activity, 10^6^ CFU/mL of *E. coli* (Gram-negative; ATCC 25922) and *S. aureus* (Gram-positive; ATCC 27853) were selected as representative microorganisms. Briefly, every sample (100 mL) was placed in 24-well plate followed by incubation with 10^6^ CFU/mL bacterial suspension for 24 h. 900 μL PBS was subsequently added to every well. Finally, 100 μL of co-cultured bacterial solution was plated on LB agar and incubated at 37°C for 24 h. The blank well was considered as a control. The test was performed triplicate under the same condition. The reduction rates of bacteria were calculated by counting the numbers of CFUs on agar plate using the following equation:


$$\text{Reduction}\;\text{rate}\;\text{of}\;\text{bacteria}\left(\%\right)=\frac{{\text{q}}_{\mathrm{control}}-{\text{q}}_{\mathrm{sample}}}{{\text{q}}_{\mathrm{control}}}\times 100\%$$

### 2.6. Cytotoxicity examinations

The extract solutions of the samples were prepared by immersing them completely in DMEM serum-free medium with a ratio of 1 mL/cm^2^ and incubated for 24 h at 37°C. After centrifugation and sterilization with a 0.22 μm filter, the extract solutions were diluted to 8 times by two-fold dilution method. Cytotoxicity on L929 fibroblast cells was evaluated using CCK-8 kit.

### 2.7. Macroscopic observation of the wound healing process

All animal experiments were approved by the Institutional Animal Care and treated humanely during the entire process. Eight-week-old BALB/c mice were generally anesthetized with 7% chloral hydrate (5 mL/kg) and then a 4 cm × 3 cm open excision type wound was created to the depth of anadesma on their backs. The wound was, respectively covered with gauze (control group), commercial Tegaderm™ diamond pattern film dressing, MQP and NQP electrospun nanofibers fixed with medical bandage. The dressings used in all groups were changed every 3 day. Photographs were taken with a digital camera to record the changes in tissue morphology of each wound at 3, 7, 10, 14 day. Wound areas were calculated by Image J software to evaluate the closure of wound. Further progression of healing with time was determined using the equation:


$$\text{Wound}\;\text{healing}\;\text{rate}\left(\%\right)=\frac{{\text{A}}_{0}-{\text{A}}_{t}}{{\text{A}}_{0}}\times 100\%$$

where A_0_ and A_*t*_ represent the wound area of first day and the wound area of post-wound days 3, 7, 10, and 14, respectively.

### 2.8. Histological examination of skin wounds

After operation for 3, 7, 14 days, the wound skin tissues were dissected. The cut tissue was fixed in 4% paraformaldehyde, dehydrated in a graded ethano and vitrified by dimethylbenzene. Then tissue was paraffin-embedded and cut into 4 μm sections for hematoxylin and eosin (H&E), Masson’s trichrome staining (MT), and CD31 immunofluorescent staining. The stained samples were observed under light microscope (Longbase 510, China).

### 2.9. Statistical analysis

All experimental data were processed using an one-way analysis of variance followed by Bonferroni’s *post-hoc* test in GraphPad Prism v8.03 (GraphPad Software Inc., San Diego, CA, USA). The data were represented as means ± standard deviations (SDs) of measurements (*p < 0.05, ^**^p < 0.01, ^***^p < 0.001, and ^****^p < 0.0001). All the experiments were repeated three times.

## 3. Results and discussion

**FIGURE 1:**
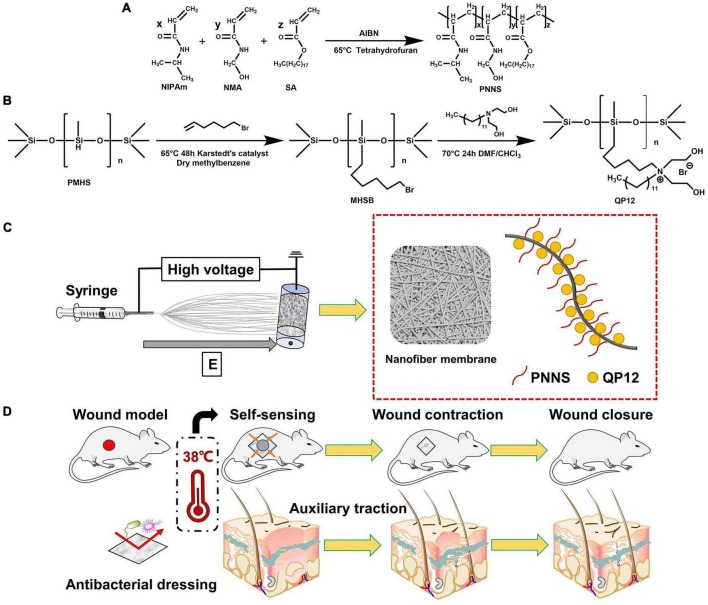
**(A)** Synthetic route of PNNS; **(B)** Synthetic route of QP12; **(C)** Schematic representation of NQP electrospun nanofibers; **(D)** Schematic diagram of the NQP electrospun nanofibers assisting wound closure by temperature-sensitive self-contraction.

Inspired by insights gained from the investigation of tissue repair mechanisms in adult and embryo, we fabricated a series of temperature-sensitive, mechanically active nanofibrous wound dressings based on QP12, PNNS, and PCL. The synthesis process of NQP electrospun nanofibers is illustrated in Figures 1A–C. Initially, the temperature-responsive polymer PNNS was synthesized. Subsequently, QP12, known for its favorable antibacterial and scar repair properties as demonstrated in our previous work, was synthesized. Lastly, QP12 and temperature-responsive PNNS was homogeneously loaded into PCL-based nanofibers using the static electrospinning technique. The resulting dressing could adhere to the wound surface and exhibit active contraction upon exposure to body temperature, thereby facilitating the contraction of the surrounding tissue (Figure 1D).

### 3.1. Synthesis and characterization of PNNS

**FIGURE 2:**
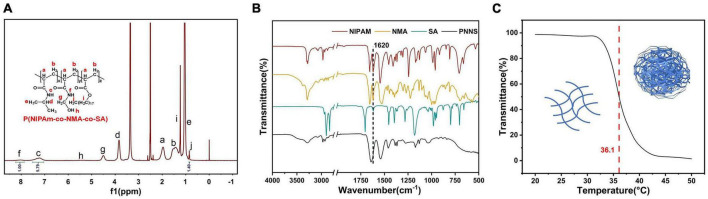
Characterization of PNNS. **(A)** The ^1^H NMR spectrum of PNNS; **(B)** The FTIR spectra of NIPAm, NMA, SA, and PNNS; **(C)** Temperature dependence of transmittance for PNNS aqueous solutions and possible conformational changes of polymers (insets).

Poly(*N*-isopropylacrylamide-co-*N*-hydroxymethylacrylamide-co-octadecyl acrylate) was synthesized through free-radical polymerization of *N*-isopropylacrylamide (NIPAm), *N*-methylol acrylamide (NMA), and octadecyl acrylate (SA). The successful synthesis of PNNS was confirmed using ^1^H NMR analysis, as shown in Figure 2A. The overlapping methylene group and methine of NIPAm and NMA protons appeared at 1.97 ppm (peak a) and 1.53 ppm (peak b), respectively. The spectrum of the NIPAm units exhibited peaks at 7.21 ppm (peak c), 3.85 ppm (peak d), and 1.04 ppm (peak e), corresponding to protons in the imino group, methine and methyl group, respectively. Notably, the main characteristic peaks of NMA units were observed at 8.03 ppm (peak f), 4.50 ppm (peak g), and 5.49 ppm (peak h), representing the imino group, methylene group and oxhydryl, respectively. Moreover, the ^1^H NMR spectrum of the SA units displayed peaks at 1.23 ppm (peak i) and 0.85 ppm (peak j), attributed to protons in the methylene and methyl groups, respectively. In the ^1^H NMR spectrum of PNNS, the characteristic peaks between 5.6 and 6.3 ppm, which corresponded to methylene protons, disappeared after the polymerization reaction. These data unequivocally demonstrated the successful synthesis of the copolymer. Similar conclusions were supported by FTIR spectra (Figure 2B). The C = C stretching peaks at 1,620 cm^–1^, present in NIPAm, NMA, and SA, were absent in PNNS, which further confirmed the formation of PNNS. Gel permeation chromatography (GPC) analysis revealed that the molecular weights (Mw and Mn) were 2,172 Da and 1,684 Da, respectively, with a molecular weight distribution (PDI) of 1.29. Additionally, PNNS exhibited thermo-responsive behavior, characterized by a reversible volume phase transition at a specific temperature known as the lower critical solution temperature (LCST). The LCST of the PNNS copolymer was determined to be 36.1°C, representing a distinct phase transition from a linear to globular conformation of the polymer chains (Figure 2C).

### 3.2. Fabrication and characterization of nanofibers

#### 3.2.1. Surface chemical structures

**FIGURE 3:**
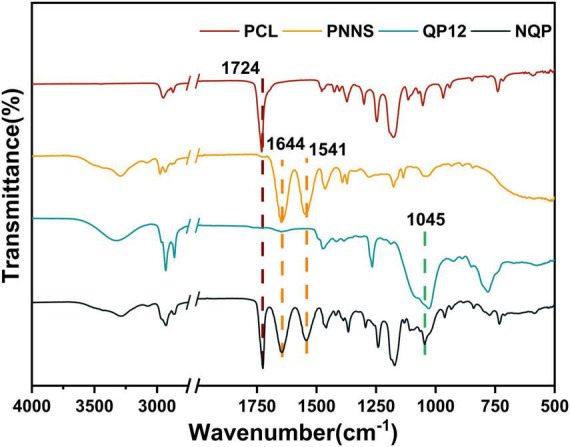
FTIR Spectra of PCL, PNNS, QP12, and NQP nanofibers.

The surface chemical structures of the nanofibers were analyzed using Fourier transform infrared transmission spectroscopy (Figure 3). In the spectra, the characteristic peaks of QP12 were observed at 1,045 cm^–1^, indicating the presence of Si-O-Si bonds. For PCL, the peaks at 1,724 cm^–1^ were attributed to C = O stretching vibrations. Additionally, the peaks at 1,644 cm^–1^ and 1,541 cm^–1^, assigned to amide I and amide II, respectively, confirmed the presence of PNNS. Moreover, the FTIR spectra of the NQP electrospun nanofibers membranes exhibited consistent signals corresponding to the different components present in the nanofibers. These findings provide evidence that the NQP electrospun nanofibers were successfully fabricated.

#### 3.2.2. Fiber morphology

**FIGURE 4:**
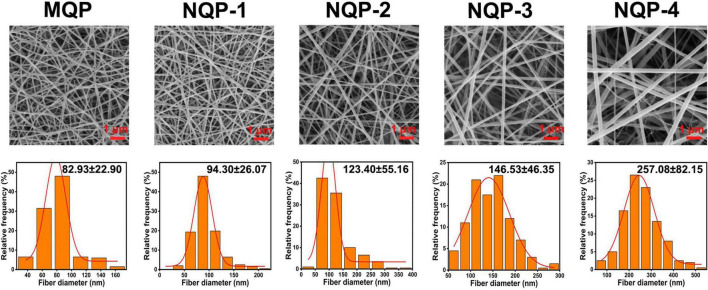
SEM images and diameter distributions of the nanofibers.

The development of dressings that closely resemble the native extracellular matrix (ECM) of skin tissue is of utmost importance in skin tissue engineering (Li et al., 2022). In this study, we employed an electrospinning technique to create four types of NQP electrospun nanofibers, namely, NQP-1, NQP-2, NQP-3, and NQP-4, with varying PNNS contents of 1, 2, 3, and 4%, respectively. SEM images and the corresponding diameter distributions of the NQP nanofibers are presented in Figure 4. The SEM images revealed that the nanofibers exhibited a homogeneous morphology, devoid of any bead-like or cracked structures, which are key characteristics of an ideal wound dressing. As the PNNS content increased, there was a corresponding increase in the diameter of the fibers, ranging from 82.93 ± 22.90 to 257.08 ± 82.15 nm. This increase in fiber diameter can be attributed to the entrapment of PNNS within the fibers (Kataria et al., 2014). Importantly, all the nanofibers exhibited diameters within the range of 50–300 nm, demonstrating a remarkable resemblance to the collagen fibrils found in the natural ECM of the skin (Parry et al., 1978).

#### 3.2.3. Water contact angles

**FIGURE 5:**
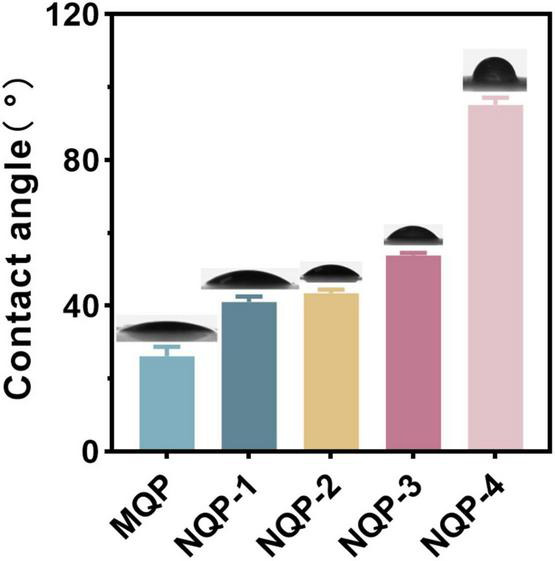
Water contact angles for the electrospun nanofibers dressing.

The wettability of nanofibers plays a crucial role in wound healing and scar suppression, and water contact angle (WCA) measurement is a reliable technique for assessing nanofiber surface wettability (Guo et al., 2020). In this study, we conducted WCA measurements to investigate the impact of PNNS content on the wettability of the nanofibers. The results demonstrated that the WCA of the nanofibers increased from 24.26 ± 0.68 to 90.74 ± 1.41 as the PNNS content increased. This increase in WCA indicated a significant decrease in the hydrophilicity of the nanofiber surface upon the addition of PNNS (Figure 5). The observed change in WCA can be attributed to the accumulation of hydrophobic PNNS on the nanofiber surface, resulting in increased water resistance.

#### 3.2.4. Water vapor transmission rate, water uptake rate, and water retention rate

**FIGURE 6:**
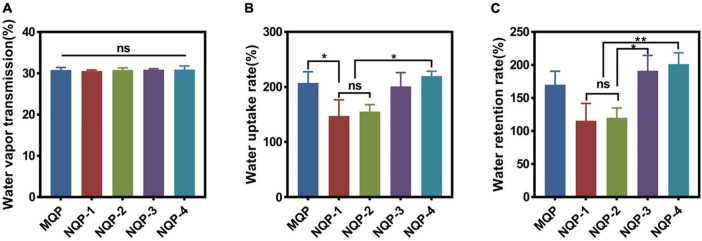
**(A)** Water vapor transmission rate (*n* = 3); **(B)** Water uptake rate (*n* = 3) and **(C)** water retention rate of the nanofibers produced (*n* = 3). *p < 0.05, **p < 0.01, ns means insignificant.

It should be noted that maintaining a moist environment is crucial for promoting active substance penetration, protecting wounds from bacterial invasion and facilitating painless removal of dressings upon recovery (Korting et al., 2011). Achieving a moist wound environment involves three key factors: water vapor transmission rate, water uptake capacity and water retention capacity. Water vapor transmission rates reflect the ability of dressing to facilitate gas exchange, preventing both dehydration and excessive exudate accumulation in wounds (Archana et al., 2013; Jayaramudu et al., 2021). Optimal water vapor transmission rates contribute to reducing replacement frequency and scar formation. Moreover, the water uptake capacity is essential for effectively absorbing excess liquid at the wound site, which can help minimize the frequency of dressing changes and reduce scar formation (Zhang et al., 2021). Additionally, the water retention capacity plays a vital role in maintaining moisture in the wound, thereby alleviating pain and enhancing wound healing capacity (Wan et al., 2020). In this study, the water vapor transmission rates, water uptake rates and water retention rates of the NQP dressings were measured and the results are presented in Figure 6. Notably, no significant differences were observed among the five types of nanofibers. The water vapor transmission rates, water uptake rates, and water retention rates fell within the required range, indicating that the NQP nanofibers have the potential to create a moist environment and effectively absorb a substantial amount of exudate, making them promising candidates for wound dressings (Yin et al., 2022).

#### 3.2.5. Mechanical properties

**FIGURE 7:**
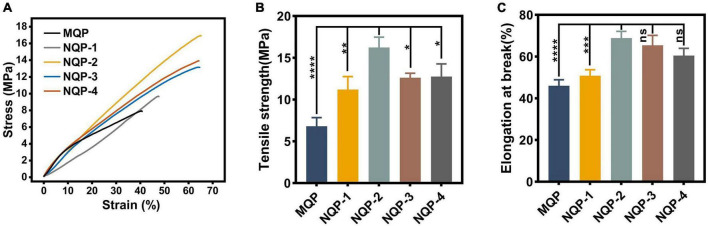
Mechanical properties of nanofibers. **(A)** Stress-strain curves; **(B)** Tensile strength (*n* = 3); **(C)** Elongation at break (*n* = 3). *p < 0.05, **p < 0.01, ***p < 0.001, ****p < 0.0001, ns means insignificant.

The mechanical properties of wound dressings play a critical role in ensuring their stability, integrity, and suitability for practical applications, thereby providing a favorable biomechanical environment during the early stages of tissue formation. The tensile strength and elongation at break are important mechanical parameters that determine the performance of nanofiber-based dressings (Fernandes et al., 2022). In normal skin, the tensile strength is typically in the range of 2.516 MPa, accompanied by an elongation at break of approximately 70% (Ma et al., 2017). In this study, the mechanical properties of the fabricated nanofibers, including tensile strength and elongation at break, were evaluated and the results are illustrated in Figure 7. It was observed that the tensile strength and elongation at break of the nanofibers increased significantly up to a PNNS concentration of 2%, primarily due to the enhanced uniformity of the nanofibers. However, when a higher content of PNNS was incorporated, both tensile strength and elongation at break showed a noticeable reduction. This finding suggests that careful consideration should be given to the PNNS concentration in order to achieve an optimal balance between mechanical strength and flexibility in the nanofiber-based wound dressings.

#### 3.2.6. Thermo-responsive properties and tissue adhesion properties

**FIGURE 8:**
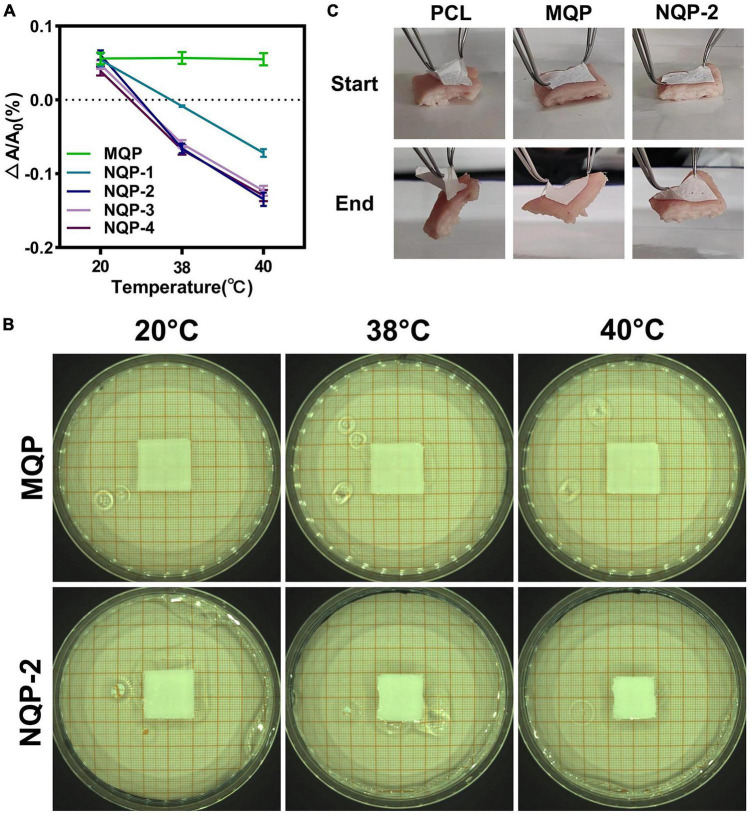
**(A)** Temperature dependent areas and **(B)** Typical optical images of NQP nanofibers immersed in 20, 38, and 40°C water; **(C)** Tissue adhesion properties of PCL nanofibers, MQP nanofibers and NQP-2 nanofibers.

The thermo-responsive property of wound dressings play a crucial role in promoting wound healing by facilitating the closure of wounds through contraction forces (Li et al., 2020). In this study, thermo-responsive nanofibers were successfully fabricated by electrospinning a solution containing PNNS polymer. The thermo-responsive behavior of the electrospun nanofibers was assessed by immersing them in distilled water at 20, 38, and 40°C, respectively. As depicted in Figures 8A, B, it was evident that the nanofiber area decreased at various temperatures, indicating the excellent temperature-sensitive contraction properties of the NQP nanofibers.

For thermo-responsive nanofibers to effectively assist wound closure, sufficient adhesion strength is of utmost importance, as it enables strong attachment to the skin and facilitates the active pulling of surrounding tissues (Qu et al., 2018). To evaluate the adhesive properties of the thermo-responsive nanofibers, porcine skin was used as a model biological tissue, and the adhesion tests were performed on the outer surface of skin. As illustrated in Figure 8C, the MQP and NQP-2 nanofibers exhibited strong tissue adhesion to the skin, primarily attributed to the adhesive properties of the silicone (Klode et al., 2011). This strong adhesion capability enhances the overall thermo-responsive performance of the nanofibers and facilitates their application in wound healing.

#### 3.2.7. Antibacterial properties and cell compatibility of nanofibers

**FIGURE 9:**
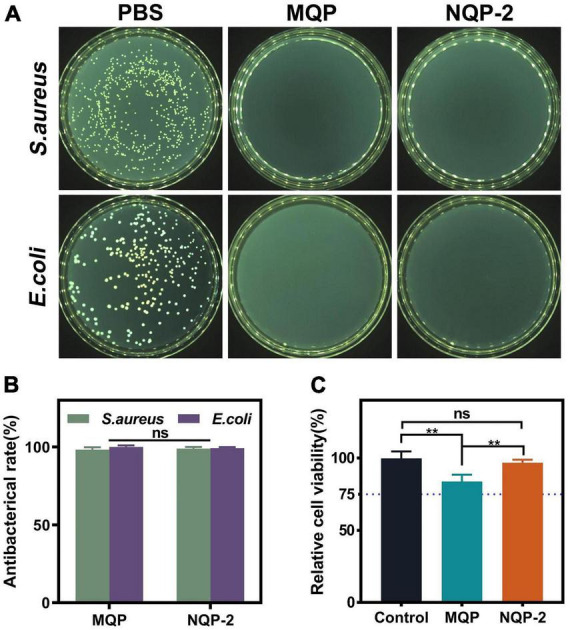
**(A)** The counter board pictures of *S. aureus* and *E. coli* growth on MQP and NQP-2 nanofibers; **(B)** Antibacterial rates of MQP and NQP-2 nanofibers (*n* = 3); **(C)** Cell viabilities of L929 fibroblast cells (*n* = 3). **p < 0.01 and ns means insignificant.

As a wound dressing, it is crucial for nanofibers to act as a barrier against external microorganisms in order to minimize the risk of infections (Mirhaj et al., 2022). The quaternized groups in QP12 possess unique antibacterial properties due to their positive charge, which enables them to bind to the negatively charged cell walls or membranes of bacteria. This interaction alters the selective permeability of bacterial cell membranes, thereby impeding nutrient uptake and ultimately leading to bacterial death (Shalel et al., 2002). To confirm the antibacterial efficacy of the QAS component, the intrinsic antibacterial properties of MQP and NQP nanofibers were assessed using *S. aureus* and *E. coli* as model organisms for Gram-positive and Gram-negative bacteria, respectively. As shown in Figure 9A, the nanofibers lacking QAS exhibited minimal inhibition of *E. coli* and *S. aureus* growth. In contrast, the presence of QAS in the nanofibers resulted in a significant reduction in bacterial colony formation. These results demonstrated that QP12 serves as the primary antibacterial component in the nanofibers. The quantitative analysis presented in Figure 9B indicated that NQP-2 nanofibers achieved nearly 99% inactivation of *E. coli* and *S. aureus*, underscoring the remarkable efficiency of NQP-2 nanofibers in eliminating both Gram-negative and Gram-positive bacteria.

Cell viability is essential for evaluating the biocompatibility of materials utilized in cell-based applications (Gharibi et al., 2021). In order to evaluate the biocompatibility of NQP-2 nanofibers, cell viabilities were examined using a leaching liquid assay with L929 fibroblast cells. As illustrated in Figure 9C, the incorporation of QP12 and PNNS led to a slight reduction in cell viabilities. However, the cell viability of NQP-2 nanofibers remained above 80% without exhibiting noticeable cytotoxic effects, thereby confirming the suitability of the selected raw materials for their intended application as wound dressings.

#### 3.2.8. *In vivo* wound closure and healing

**FIGURE 10:**
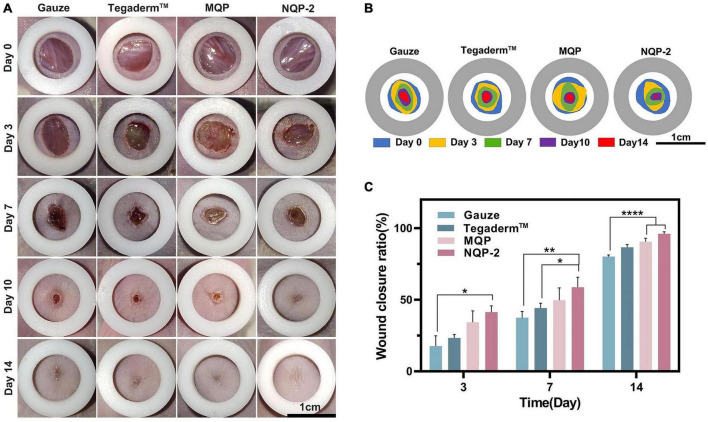
**(A)** Images of the wound healing sites on days 0, 3, 7,10, and 14; **(B)** Traces of wound-bed closure on days 0, 3, 7, 10, and 14; **(C)** Quantitative analysis of wound healing rates according to panel **(A)** (*n* = 3). *P < 0.05, **p < 0.01, and ****p < 0.0001.

The potential wound healing efficacy of NQP-2 nanofibers in wound healing was evaluated using a full-thickness skin defect model. Figures 10A–C shows the wound contraction of different treatments, including gauze, Tegaderm™, MQP, and NQP-2 nanofibers, at various time points (day 0, 3, 7, 10, and 14). Remarkably, NQP-2 nanofibers exhibited a significant enhancement in wound closure, accelerated wound healing and reduced scarring compared to the other groups. During the early stage of wound healing, the control group exhibited persistent pus at the wound site, whereas the nanofiber-treated group demonstrated initial signs of healing. This disparity could be attributed to the superior antibacterial properties of the nanofibers, which facilitated early scab formation. Moreover, the wound contraction area of the groups treated with NQP-2 nanofibers surpassed that of nanofibers lacking the contraction ability. This result indicated that the thermo-responsive self-contraction capability of the nanofibers could provide the necessary contraction force to assist in the closure of gaping wounds. By day 14, only the wounds treated with NQP-2 nanofibers displayed complete closure, while the other groups exhibited unhealed wounds with hypertrophic scars and visible erythema. These results suggest that NQP-2 nanofibers can facilitate wound closure and accelerate the process of wound healing.

**FIGURE 11:**
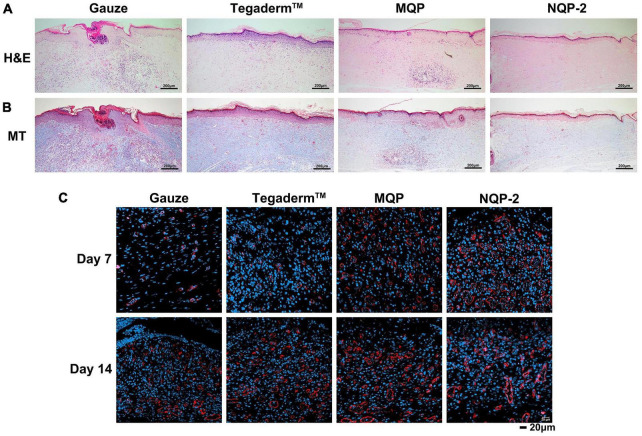
**(A)** H&E staining of wound sections at day 14; **(B)** Masson’s staining of wound sections at day 14; **(C)** Immunofluorescence staining of CD31 structures at days 7 and 14.

To further assess the wound-healing process, histological analysis was conducted and the results are presented in Figure 11. Figure 11A demonstrates the tissues treated with gauze, Tegaderm™, MQP, and NQP-2 nanofibers on day 14, which were stained with hematoxylin and eosin (H&E). We found that the NQP-2 nanofiber treatment exhibited the most efficient wound healing, as indicated by fewer inflammatory cells, increased neovascularization, enhanced fibroblast generation and hair follicle development. In contrast, the gauze-treated group displayed severe wound opening, indicating the initial stages of the healing process. Compared to the Tegaderm™ and MQP groups, the NQP-2 nanofiber-treated group exhibited reduced inflammatory cell infiltration and higher distribution of neovascularization and fibroblasts.

Furthermore, to investigate the promotion biological mechanisms and evaluate the application of NQP-2 nanofibers in wound healing, Masson’s trichrome staining (MT) and CD31 immunofluorescence staining were performed to examine the collagen deposition and angiogenesis throughout wound healing process. As depicted in Figure 11B, the NQP-2 nanofiber-treated group exhibited increased collagen deposition within the wound bed and displayed excellent collagen alignment. Moreover, the immunofluorescence staining of CD31 in Figure 11C revealed significantly higher expression of CD31 protein in the NQP-2 group compared to that of other groups, suggesting that NQP-2 nanofibers can enhance angiogenesis and promote wound healing.

## 4. Conclusion

In summary, the advent of wound-contractible dressings holds great promise for improving patient outcomes. The combination of temperature-sensitive therapy with wound dressings represents a promising direction for the development of a new generation of wound dressings. By incorporating temperature-sensitive PNNS into PCL-based electrospun nanofibers, we have successfully engineered a wound-contractible dressing capable of facilitating wound closure and promoting the healing process. Additionally, the inclusion of QP12 has enhanced the antibacterial and anti-scarring properties of the nanofibers. The fabricated electrospun nanofibers actively induce wound contraction, alleviate inflammation, mitigate scar formation and improve the overall quality of wound healing. Our wound dressing, developed based on embryonic wound healing, counteracts some of the tension in the wound and reduces the effect of external tension on the wound, which helps to inhibit inflammation and scarring. These features indicate that the multifunctional nanofibers are efficient and versatile for many kinds of wound healing, positioning them as promising candidates for skin wound repair.

## Data availability statement

The original contributions presented in this study are included in this article/supplementary material, further inquiries can be directed to the corresponding authors.

## Ethics statement

The animal study was reviewed and approved by the Animal Ethics Committee of Binzhou Medical University.

## Author contributions

GH conceived and designed the experiments. QQ completed the polymer purification and antimicrobial assays, and collaborated with RL on other parts of the experiments. CL performed the data analysis. CL and QQ wrote the manuscript. CW supervised the work and revised the manuscript. All authors contributed to the article and approved the submitted version.
